# S1-R2 and R1-R2 Backward Crosstalk Both Affect the Central Processing Stage

**DOI:** 10.5334/joc.121

**Published:** 2020-10-06

**Authors:** Valentin Koob, Moritz Durst, Daniel Bratzke, Rolf Ulrich, Markus Janczyk

**Affiliations:** 1 Department of Psychology, University of Bremen, Bremen, DE; 2 Department of Psychology, Eberhard Karls University of Tübingen, Tübingen, DE

**Keywords:** Dual-task, Backward Crosstalk, Psychological Refractory Period, Compatibility

## Abstract

A frequent observation in dual-task experiments is that performance in Task 1 is influenced by conceptual or spatial overlap with features of Task 2. Such compatibility-based backward crosstalk effects (BCEs) can occur when overlap exists between the responses of two tasks–the R1-R2 BCE–or between the stimulus in Task 1 and the response in Task 2–the S1-R2 BCE. The present study investigated whether the S1-R2 BCE has a perceptual locus, and by implication, whether the two BCEs have a common processing locus or different ones. To this end, we applied the additive factors logic and manipulated the duration of the Task 1 perceptual stage. The results argue against a perceptual locus for both BCEs. As a possible explanation, we suggest that the R1-R2 BCE and the S1-R2 BCE have their locus within a capacity-limited central stage, but that they arise from different processes within this stage. The R1-R2 BCE influences Task 1 response selection, whereas the S1-R2 BCE influences Task 1 stimulus classification. A plausible though post-hoc model is presented within the Discussion.

## Introduction

Having to deal with multiple tasks at hand (i.e., multitasking) is typical rather than the exception in daily life. Although psychological research generally agrees in that multitasking often comes with performance decrements (e.g., Kiesel et al., 2010; Koch et al., 2018; but see Brüning et al., in press), there is little consensus about the precise way concurrent tasks are processed. In particular, a central and ongoing debate concerns whether humans can perform two tasks simultaneously (e.g., Meyer & Kieras, 1997; Miller et al., 2009; Navon & Miller, 2002; Tombu & Jolicœur, 2003) or whether certain parts of both tasks can only be carried out one after another in a serial fashion. While generally perceptual and motor stages of two tasks might run in parallel to any other processing stage, the central stage can only process one task at any point in time according to the latter view (Pashler, 1994; Welford, 1952). The second of two subsequently performed tasks must wait to gain access to this capacity-limited stage (for exceptions, see Janczyk, Pfister, Wallmeier, & Kunde, 2014), and this idle time of waiting is known as the cognitive slack. The often-deployed *response selection bottleneck* (RSB) model (Pashler, 1994; Welford, 1952) assumes that the serial capacity-limited central stage concerns response selection, thus limiting the cognitive system to apply only one stimulus-response rule (S-R) at a time (see Janczyk & Kunde, 2020, for an alternative interpretation).

Nevertheless, evidence contradicting the notion of strictly serial processing has accumulated recently. A particularly interesting piece of evidence in this regard is the backward crosstalk effect (BCE), that is, the observation that certain aspects of Task 2 already influence performance in Task 1 (Ellenbogen & Meiran, 2008; Giammarco et al., 2016; Hommel, 1998; Janczyk, 2016; Janczyk, Pfister, Hommel, & Kunde, 2014; Janczyk et al., 2018; Ko & Miller, 2014; Lien & Proctor, 2002; Logan & Delheimer, 2001; Logan & Schulkind, 2000; Miller, 2006). Processes related to response selection of the two tasks are thus not entirely isolated from each other. The exact mechanism, however, is highly debated and largely depends on the specific characteristics of the tasks at hand; such as working memory demands (e.g., Ellenbogen & Meiran, 2008; Hommel & Eglau, 2002), overlap at the response and/or stimulus level (e.g., Rieger & Miller, 2020), type of backward crosstalk (e.g., Durst & Janczyk, 2019), or the context of practice (e.g., Giammarco et al., 2016).

### Two types of compatibility-based BCEs

A compatibility-based BCE was first reported by Hommel (1998), who observed that even response times in Task 1 (RT1s) were shorter when certain task features of Task 1 were compatible with features of Task 2. In his first experiment, participants were presented with colored letters. The color of the letter required a manual left versus right keypress, whereas the identity of the letter was answered by vocally uttering the word ‘left’ versus ‘right’ (originally in German). The tasks thus had a (spatial) overlap in terms of their responses. When both responses were spatially compatible (e.g., a left manual keypress and a vocal ‘left’ utterance), RT1s were shorter than when both responses were spatially incompatible (e.g., a left manual keypress and a vocal ‘right’ utterance). This type of compatibility-based crosstalk, which we will henceforth refer to as the R1-R2 BCE, was replicated in numerous subsequent studies using two manual responses (Janczyk, Pfister, Hommel, & Kunde, 2014; Lien & Proctor, 2000; Miller & Durst, 2014; Thomson et al., 2010; Thomson et al., 2015; Watter & Logan, 2006), manual and pedal responses (Durst & Janczyk, 2019; Janczyk, 2016), or manual and vocal responses (Ellenbogen & Meiran, 2011; Renas et al., 2018).

Yet, a compatibility-based BCE can also be obtained when the stimulus in Task 1 (S1) conceptually overlaps with the response in Task 2 (R2) – the S1-R2 BCE. In his second Experiment, Hommel (1998) changed the instructions for Task 2. Participants now responded to the identity of the letter with the vocal utterance ‘red’ versus ‘green’ (originally in German). This created an overlap between S1 (red or green) and the response for Task 2 (uttering ‘red’ or ‘green’). When S1 and R2 were conceptually compatible (e.g., a red letter and the utterance ‘red’), RT1s were shorter relative to when S1 and R2 were conceptually incompatible (e.g., a red letter and the utterance ‘green’). Although investigated in one of the very first studies, the S1-R2 BCE has yet received relatively little attention in the literature. Only a few other studies reported instances of S1-R2 BCEs with conceptual overlap between the color of S1 and a vocal R2 (Ellenbogen & Meiran, 2008; Hommel & Eglau, 2002) and spatial overlap between an auditory S1 and a manual R2 (Lien et al., 2007). In sum, at least two types of compatibility-based BCEs can be distinguished: the R1-R2 BCE depends on the overlap between two responses, whereas the S1-R2 BCE depends on the conceptual overlap between S1 and R2 (for another type of compatibility-based BCE, see Rieger & Miller, 2020).

### Theoretical explanations for compatibility-based BCEs

Conceivably, parts of the Task 2 response must be activated while Task 1 processing is still ongoing to yield a compatibility-based BCE. To account for this, several authors made the additional assumption of a response activation stage in between the stages of perception and response selection. Response activation itself is considered capacity-unlimited and thus can run in parallel with all other stages of another task (Hommel, 1998; Lien & Proctor, 2002; Schubert et al., 2008). When the temporal overlap of Task 1 and Task 2 is sufficiently high (i.e., at a short stimulus onset asynchrony, SOA), response activation from Task 2 can influence that of Task 1 (and vice versa). The result is a lengthened Task 1 response activation stage in incompatible relative to compatible trials, expressing itself in the form of a BCE (Hommel, 1998). According to this view, response activation is the *locus* of compatibility-based BCEs.

In contrast, recent studies identified the locus of the R1-R2 compatibility-based BCE in the capacity-limited central stage of Task 1 (Janczyk et al., 2018; Thomson et al., 2015). It is argued that response selection and response activation are not separate stages which run one after another. Rather, given that Task 1 has direct access to the central stage, the translation of S1 into a corresponding response in Task 1, usually denoted as response selection, starts immediately after stimulus perception (see Figure 1c of Janczyk et al., 2018). Task 1 response activation might still occur, but it is not a separate stage, distinct from and prior to Task 1 response selection. For Task 2, however, a different picture emerges. Response selection of Task 2 is delayed by Task 1, separating the process of response activation from the controlled one of response selection. As such, Task 2 response activation immediately follows stimulus perception and runs in parallel to Task 1 processing for some time. When Task 2 response activation temporally overlaps with the central stage of Task 1 response selection, crosstalk between both tasks is enabled and might influence the duration of the capacity-limited central stage of Task 1 (which then constitutes the *locus*).

**Figure 1:**
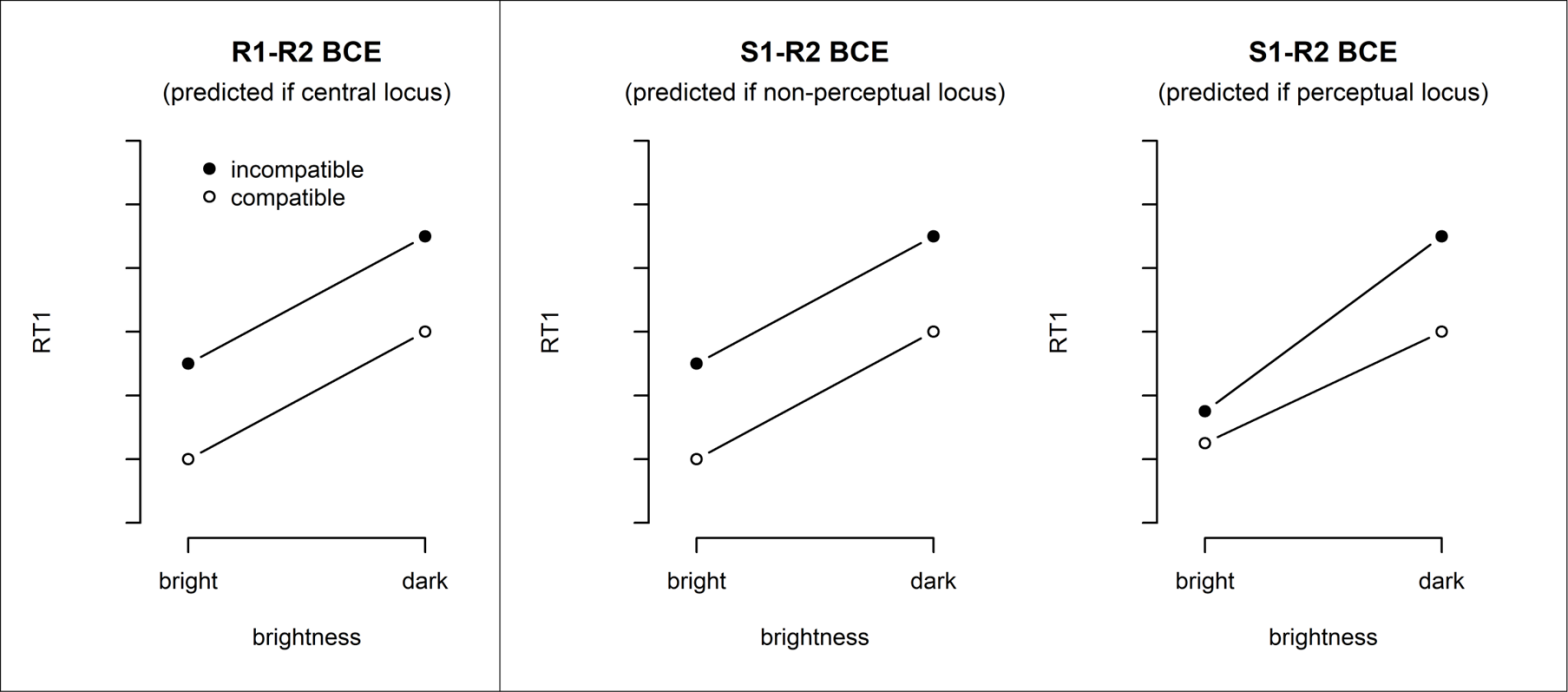
Idealized predictions for the R1-R2 and the S1-R2 BCE as a function of stimulus brightness. The R1-R2 BCE is assumed to have its locus within the capacity-limited central stage and should thus not interact with stimulus brightness (left panel). For the S1-R2 BCE, two options are conceivable (middle and right panel). If the S1-R2 BCE has its locus outside the perceptual stage, a pattern similar to the R1-R2 BCE should emerge (middle panel). If, however, the S1-R2 BCE has its locus within the perceptual stage, some kind of interaction with stimulus brightness should emerge (right panel).

Regarding the S1-R2 BCE, crosstalk is established between perceptual features of Task 1 and response features of Task 2. One might thus assume that the S1-R2 BCE has its locus within the perceptual stage of Task 1. Important for the current study, Janczyk et al. (2018), however, obtained some evidence in their third experiment that the S1-R2 BCE has its locus within the capacity-limited processing stage as well – similar to the R1-R2 BCE. Yet, the results nevertheless exhibited a descriptive trend that would be consistent with a perceptual locus of the S1-R2 BCE.^1^ Thus, at the present state, we cannot unambiguously exclude the involvement of the perceptual stage within the S1-R2 BCE, and–to the best of our knowledge–no other study has yet explicitly addressed this issue.

### The present study

Against this background, the present experiment aims to clarify whether the S1-R2 BCE has its locus in the perceptual stage or not. If not, we then might assume that the S1-R2 BCE has its locus in the capacity-limited central stage instead and may thus share its locus with the R1-R2 BCE. To anticipate, this is what we conclude from our results.

To this end, participants performed separate blocks of dual-task trials, in which either an R1-R2 BCE or an S1-R2 BCE was enabled. In both types of blocks, we varied the brightness of S1 on a trial-by-trial basis – a manipulation affecting the perceptual stage (see Pashler & Johnston, 1989). We expected shorter RT1s for bright compared to dark S1s, and R1-R2 and S1-R2 BCEs in the respective blocks. The crucial predictions rely on Sternberg’s (1969) additive factors logic, which predicts an interaction of two experimental factors if they affect the same processing stage. If they affect different processing stages, on the other hand, additive effects are expected. As there is available evidence suggesting that the R1-R2 BCE has its locus in the central stage (Janczyk et al., 2018; Thomson et al., 2015), it should yield an additive effect of the R1-R2 BCE with S1 brightness. For the S1-R2 BCE, however, the trend towards underadditivity in Experiment 3 of Janczyk et al. (2018) leaves open the possibility of a perceptual locus. In this case, an interaction of the S1-R2 BCE with S1 brightness is expected. Additionally, because one would expect an interaction in blocks with the S1-R2 BCE, but not in blocks with the R1-R2 BCE, a three-way interaction between S1 brightness and the two types of BCEs should be present as well. In contrast, if the S1-R2 BCE does not have its locus in the perceptual stage, the S1-R2 BCE should have an additive effect with S1 brightness, and the three-way interaction should not be significant (see Figure 1 for a visualization of the predicted RT patterns). Since this required retaining null hypotheses, we additionally employed a Bayesian approach to data analyses.

## Method

### Participants

Forty-eight students (34 female) from the University of Tübingen, aged 19 to 45 years (*M* = 23.6 years, *SD* = 5.11), participated for monetary compensation (8€) or course credit. All participants provided written informed consent before the experiment and had normal or corrected-to-normal vision.

### Apparatus and stimuli

A standard PC was used for stimulus presentation and response collection. Stimuli and instructions were presented on a 17-in. CRT monitor. Stimuli were red or green colored frames surrounding the white letters ‘H’ or ‘S’, presented in the center of an otherwise black screen. The color of the frame served as S1 and was either bright or dark. The identity of the letter served as S2. R1s were given manually via two response keys, one to the left and one to the right of the participant. R2s were vocal utterances. RT2s were measured with a voice key and the experimenter entered the identity of the response immediately.

### Tasks and procedure

**Figure 2:**
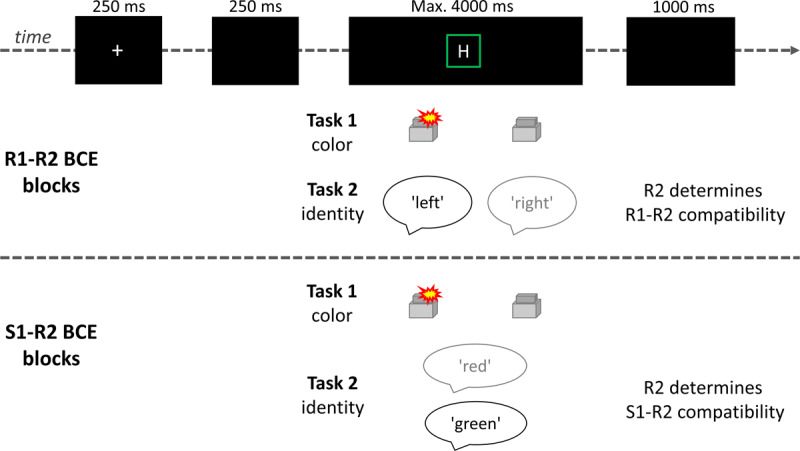
Trial structure and tasks of the two different block types. In each trial, participants first responded to the color of the frame in a manual two-choice task by pressing a left or right key (Task 1). Then, participants responded to the identity of the letter in a vocal two-choice task (Task 2). In R1-R2 BCE blocks, the responses were the words ‘left’ and ‘right’, and in S1-R2 BCE blocks, they were ‘red’ and ‘green’. In the depicted example, Stimulus 1 is a green frame that requires a left index finger response in Task 1. In Task 2 of R1-R2 BCE blocks, the identity ‘H’ requires a vocal utterance of the word ‘left’, while the letter ‘S’ indicates a ‘right’ utterance. In this trial, the R1-R2 relation is compatible. In Task 2 of S1-R2 BCE blocks, the identity ‘H’ requires a vocal utterance of the word ‘green’, while the letter ‘S’ indicates a ‘red’ utterance. In this trial, the S1-R2 relation is compatible.

The trial structure and tasks are illustrated in Figure 2. Task 1 (color) was to respond to S1 with a manual key press of the left or right index finger (R1), and Task 2 (letter identity) was to respond to S2 with a vocal utterance (R2). In R1-R2 BCE blocks, R2 was the vocal utterance ‘links’ or ‘rechts’ (German for ‘left’ and ‘right’). In S1-R2 BCE blocks, R2 was the vocal utterance ‘rot’ or ‘grün’ (German for ‘red’ and ‘green’). The S-R mappings of both tasks were counterbalanced across participants.

Each trial started with a white fixation cross (250 ms), followed by a blank screen (250 ms). Then, a bright or dark-colored frame surrounding the letter was presented at the center of the screen. Frame and letter were displayed at the same time (i.e., the SOA was 0 ms) for a maximum of 4000 ms or until both responses were registered. The next trial started after an inter-trial interval (ITI) of 1000 ms. In case of an error, a respective feedback message was presented on the screen for 1000 ms before the ITI.

Half of the participants started with the R1-R2 BCE task blocks, the other half with the S1-R2 BCE task blocks. Participants first performed a short practice block of 20 randomly drawn trials of the respective BCE task, followed by six experimental blocks of 64 trials, resulting from eight repetitions of all combinations of 2 S1 (red or green frame) × 2 S2 (‘H’ or ‘S’) × 2 S1 brightness (bright or dark). All trials were presented in a random order within each half of the experiment. Participants received written instructions that emphasized speed as well as accuracy and were asked to give R1 and R2 successively in a fixed order.

### Design and analysis

In R1-R2 BCE blocks, trials in which the required spatial position of R1 and the required vocal R2 matched (e.g., left index finger and the utterance ‘links’) were R1-R2 compatible, whereas the other trials were R1-R2 incompatible. In S1-R2 BCE blocks, trials in which the color of S1 and the required vocal R2 matched (e.g., a red frame and the utterance ‘rot’) were S1-R2 compatible, whereas the other trials were S1-R2 incompatible. Data from practice blocks, trials with task-unspecific errors (missing responses, wrong response order, two responses in one task), and trials with an inter-response interval (IRI) of less than or equal to 100 ms were excluded.^2^ For the analysis of RTs, only trials with correct responses in both tasks were considered. From the remaining ones, trials deviating more than 2.5 standard deviations from the individual cell mean were considered as outliers and excluded from the analysis (2.81% and 2.68% for Task 1 and Task 2, respectively). Mean RTs and error rates (ERs) were then submitted to separate 2 × 2 × 2 Analyses of Variances (ANOVAs) with the within-subject factors (1) compatibility (compatible vs. incompatible), (2) block type (R1-R2 BCE vs. S1-R2 BCE), and (3) S1 brightness (bright vs. dark). As mentioned before, our primary focus was on the significance/non-significance of the interaction terms compatibility × brightness and compatibility × block type × brightness. Because traditional ANOVA cannot assess evidence for the null hypotheses, we additionally calculated Bayes Factors for the two theoretically relevant interactions. Bayes Factors compare the probabilities of data given one model over another (e.g., interaction absent vs. interaction present). When multiplying a Bayes Factor with prior beliefs about each model, one can calculate a ratio that expresses which model is more likely given the data. As such, they provide a measure of the relative strength of evidence favoring either one of the compared models (see, e.g., Masson, 2011). We calculated Bayes Factors by comparing the full ANOVA-model (as the denominator) with models leaving the respective terms out, using the *Bayes Factor* package in R (Morey & Rouder, 2018).

## Results

### Task 1

**Figure 3:**
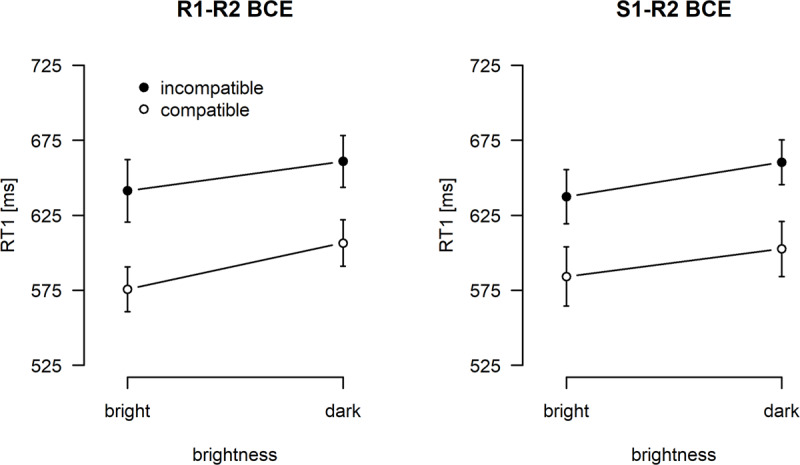
Task 1 response times (RT1; in milliseconds) as a function of compatibility and brightness, displayed separately for each block type (R1-R2 BCE: left panel, S1-R2 BCE: right panel). Error bars indicate 95% confidence intervals after removing inter-subject variability.

Mean RT1s are visualized in Figure 3 (see also Table 1). Regarding the main effects, responses were faster in compatible (592 ms) relative to incompatible (650 ms) trials, the classical BCE, *F*(1,47) = 50.91, *p* < .001, \eta _{\rm{p}}^2 = .52, and when S1 was bright (610 ms) compared to dark (633 ms), *F*(1, 47) = 67.50, *p* < .001, \eta _{\rm{p}}^2 = .59. Neither the main effect of block type, nor any of the interactions reached statistical significance; block type, *F*(1, 47) < 0.01, *p* = .999, \eta _{\rm{p}}^2 < .01; compatibility × block type, *F*(1, 47) = 0.11, *p* = .746, \eta _{\rm{p}}^2 < .01; compatibility × brightness, *F*(1, 47) = 0.27, *p* = .605, \eta _{\rm{p}}^2 = .01; block type × brightness, *F*(1, 47) = 0.96, *p* = .333, \eta _{\rm{p}}^2 = .02; compatibility × block type × brightness, *F*(1, 47) = 1.99, *p* = .165, \eta _{\rm{p}}^2 = .04. Most important, however, these results included a non-significant interaction of compatibility with brightness, as well as a non-significant three-way interaction between compatibility, brightness, and block type. In both cases, the Bayes Factors indicated a slight tendency in favor of the null hypotheses; compatibility × brightness, B_01_ = 6.2 (±1.0%), compatibility × block type × brightness, B_01_ = 4.1 (±0.7%).

**Table 1:** Task 1 and Task 2 Mean Response Times and Error Rates as a Function of Compatibility, Brightness, and Block Type.

	Block type	Brightness	Task 1			Task 2		

			Compatibility			Compatibility		

			Incompatible	Compatible	Δ	Incompatible	Compatible	Δ

RT								
	R1-R2	Bright	641	576	65	1174	1090	84
		Dark	661	607	54	1204	1123	81
	S1-R2	Bright	637	584	53	1228	1138	90
		Dark	660	603	57	1257	1158	99
ER								
	R1-R2	Bright	6.3	2.0	4.3	4.4	2.6	1.8
		Dark	6.9	3.2	3.7	4.1	2.6	1.5
	S1-R2	Bright	4.2	3.1	1.1	4.2	2.3	1.9
		Dark	4.5	3.5	1.0	4.7	2.5	2.2

*Note*: RT = response time; ER = error rate; Δ = difference between compatible and incompatible trials, resembling the crosstalk effects within the respective conditions.

Mean ERs in Task 1 are summarized in Table 1. First, participants made more errors when S1 was dark (4.5%) compared to bright (3.9%), *F*(1, 47) = 6.18, *p* = .017, \eta _{\rm{p}}^2 = .12. Second, ERs were higher in the R1-R2 (4.6%) relative to the S1-R2 (3.8%) blocks, *F*(1, 47) = 7.25, *p* = .010, \eta _{\rm{p}}^2 = .13. Third, ERs were higher in incompatible (5.5%) relative to compatible (2.9%) trials, *F*(1, 47) = 32.38, *p* < .001, \eta _{\rm{p}}^2 = .41. This difference was much smaller in the S1-R2 (4.4% vs. 3.3%) than in the R1-R2 (6.6% vs. 2.6%) blocks, as reflected by a significant interaction of compatibility with block type, *F*(1, 47) = 7.36, *p* = .009, \eta _{\rm{p}}^2 = .14. Most important, however, all other interactions did not reach statistical significance; compatibility × brightness, *F*(1, 47) = 0.48, *p* = .491, \eta _{\rm{p}}^2 = .01; block type × brightness, *F*(1, 47) = 1.85, *p* = .180, \eta _{\rm{p}}^2 = .04; compatibility × block type × brightness, *F*(1, 47) = 0.38, *p* = .543, \eta _{\rm{p}}^2 = .01. This (again) included a non-significant interaction of compatibility with brightness, as well as a non-significant three-way-interaction between compatibility, block type, and brightness. The according Bayes Factors for both interactions indicated a slight preference for the null hypotheses, B_01_ = 6.0 (±1.8%), and B_01_ = 4.4 (±1.4%), respectively.

### Task 2

Mean RT2s are summarized in Table 1, with all main effects reaching statistical significance. First, participants responded faster in compatible (1127 ms) relative to incompatible (1216 ms) trials, *F*(1, 47) = 70.42, *p* < .001, \eta _{\rm{p}}^2 = .60. Second, RT2s were shorter when S1 was bright (1157 ms) compared to dark (1186 ms), *F*(1, 47) = 58.78, *p* < .001, \eta _{\rm{p}}^2 = .56, likely resulting from propagation of the corresponding Task 1 effect. Third, RT2s were shorter in the R1-R2 (1148 ms) compared to the S1-R2 (1195 ms) blocks, *F*(1, 47) = 5.21, *p* = .027, \eta _{\rm{p}}^2 = .10. Importantly, as for RT1, none of the interaction terms reached statistical significance; compatibility × block type, *F*(1, 47) = 0.46, *p* = .499, \eta _{\rm{p}}^2 = .01; compatibility × brightness, *F*(1, 47) = 0.12, *p* = .729, \eta _{\rm{p}}^2 < .01; block type × brightness, *F*(1, 47) = 0.99, *p* = .325, \eta _{\rm{p}}^2 = .02; compatibility × block type × brightness, *F*(1, 47) = 0.59, *p* = .445, \eta _{\rm{p}}^2 = .01. The according Bayes Factors for the two-way interaction of compatibility with brightness as well as for the three-way interaction of compatibility, block type, and brightness indicated a slight preference for the null hypotheses, B_01_ = 6.4 (±1.6%), and, B_01_ = 4.5 (±1.4%), respectively.

Errors in Task 2 (see Table 1) occurred more often in incompatible (4.3%) relative to compatible (2.5%) trials, *F*(1, 47) = 25.66, *p* < .001, \eta _{\rm{p}}^2 = .35. Neither one of the remaining main effects nor any interaction reached statistical significance, *F*s(1, 47) ≤ 1.65, *p*s ≥ .206, \eta _{\rm{p}}^2 \le .03. The according Bayes Factors for the two-way interaction of compatibility with brightness and for the three-way interaction between compatibility, brightness and block type indicated a slight preference for the null hypotheses, B_01_ = 6.4 (±1.9%), and, B_01_ = 4.0 (±1.4%), respectively.

## Discussion

Within the dual-tasking literature, an important line of research indicates that Task 1 processing is prone to interference from Task 2 response activation. Specifically, in the case of a conceptual or spatial overlap between features of Task 1 and Task 2, RT1s are often shorter when these features are compatible relative to incompatible, the so-called (compatibility-based) backward crosstalk effect (BCE; e.g., Durst & Janczyk, 2019; Hommel, 1998; Janczyk, Pfister, Hommel, & Kunde, 2014; Lien et al., 2007; Watter & Logan, 2006). Compatibility can be defined by (spatial) overlap of two responses, the R1-R2 BCE, but also by conceptual overlap of S1 and R2, the S1-R2 BCE. In the present study, we aimed to enhance our knowledge about–in particular–the S1-R2 BCE by investigating whether it has its locus within the perceptual stage of Task 1 or not.

### S1-R2 and R1-R2 crosstalk effects and their influence on Task 1 processing

As described in the introduction, Task 2 response activation in the context of the R1-R2 BCE likely affects the duration of the capacity-limited central stage of Task 1, that is, the stage of response selection (Janczyk et al., 2018; Thomson et al., 2015). The S1-R2 BCE, on the other hand, expresses interference between perceptual features of S1 and response characteristics of R2. One obvious way to think about this type of BCE is thus that it affects the perceptual stage of Task 1. To test this, we manipulated the Task 1 perceptual stage by presenting either dark or bright S1 stimuli. Following Sternberg’s (1969) additive factors logic, we should observe an interaction between stimulus brightness and S1-R2 compatibility, if S1-R2 crosstalk affects the same Task 1 perceptual stage as our perceptual manipulation. If not, an additive effect is expected. In sum, we did not observe convincing statistical evidence for an interaction, neither in terms of RTs nor in terms of ERs, as was further indicated by Bayesian inference.^3^ Our results are straightforward and render a perceptual locus of the S1-R2 BCE unlikely. In conjunction with Experiment 3 of Janczyk et al. (2018), we thus believe that such interference targets the capacity-limited central stage, similar to the R1-R2 BCE. In this latter experiment, a descriptive underadditive interaction between the S1-R2 BCE and an SOA manipulation was observed (see the Introduction for further information), which would be consistent with a pre-central locus of the S1-R2 BCE. However, since the interaction was not statistically significant the authors argued against a pre-central locus. The present results support this conclusion.

### A possible mechanism underlying the R1-R2 and S1-R2 compatibility effect

**Figure 4:**
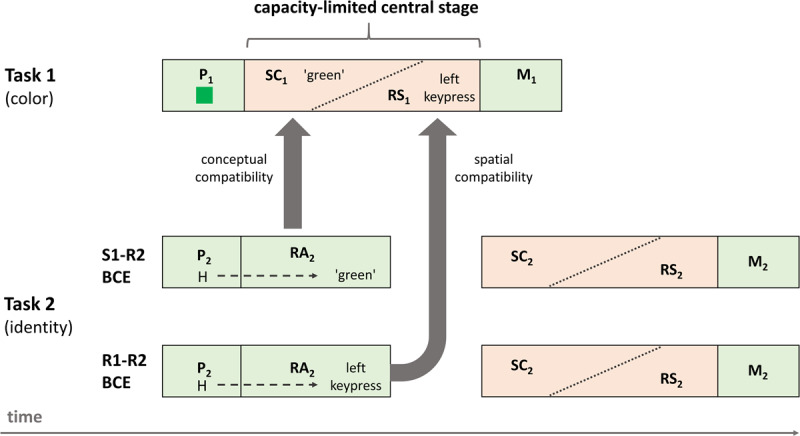
Illustration of a tentative model for the S1-R2 and the R1-R2 BCE. The capacity-limited central stage (orange) comprises two (maybe cascaded) processes separated by a diagonal and dotted line. Task 2 (the identity task) is illustrated separately for the S1-R2 BCE and the R1-R2 BCE. Note that the stimuli and responses shown here are taken from Experiment 1 (R1-R2 BCE) and 2 (S1-R2 BCE) of Hommel (1998). The model assumes that the locus of the S1-R2 BCE is in the capacity-limited process of stimulus classification (SC), whereas the locus of the R1-R2 BCE is in the capacity-limited process of response selection (RS). Grey arrows indicate the source of the two BCEs in response activation (RA) of Task 2. (P = perceptual stage, M = motor stage, subscripts indicate Task 1 and 2, respectively).

Based on the results, we suggest a tentative model integrating both BCEs in the following. Of course, this model is not tested with the present data, but we believe that it is reasonable and might guide future research when theorizing about compatibility-based BCEs (see Figure 4).

Consider a basic model of task processing comprising (at least) four processes to transform a stimulus into a required response (see also Johnston & McCann, 2006; Sanders, 1980): stimulus perception, stimulus classification, response selection, and motor production (see Figure 4). Stimulus classification is understood as meaning that a perceived stimulus with its modal features (e.g., the red-ness of a frame) is ‘linked’ to a semantic category (e.g., an amodal representation of RED that may be linked to the red color, but also to perceiving the word ‘red’). Then, a process of response selection links the category to a certain response via a category-response (C-R) rule (e.g., if RED then press left key; see also, e.g., Fagot & Pashler, 1992, for a related view). Assuming such a two-step process seems reasonable for two reasons. First, recent evidence indicates that the BCE is (at least partially) mediated via category-response (C-R) rules (Ellenbogen & Meiran, 2008; Thomson et al., 2010), and not necessarily via stimulus-response rules (Hommel, 1998).^4^ Second, for an S1-R2 BCE to occur, S1 and R2 need to overlap. Such an overlap would be hard to imagine if stimulus transition was merely based on S-R rules. This is because (a) we excluded a perceptual locus and (b) the output of such a translation process (e.g., a left response for S1) does not overlap with R2 (e.g., a ‘green’ utterance for S2). Instead, assuming an overlap at the level of semantic categories would inherently solve this issue, even though the category might only incorporate a single exemplar (e.g., the same ‘red’/‘green’ or ‘H’/‘S’ stimuli).

While capacity-limitations for response selection are usually taken for granted, the same argument has been made for stimulus classification (Johnston & McCann, 2006). Thus, we suggest that both processes occur during the capacity-limited central stage but need not necessarily be seen as distinct and serial stages. Instead, it might well be that stimulus classification and response selection are cascaded and hence do not run entirely one after the other in a strictly serial way (see, e.g., Dell, 1986, for a cascade model of speech production). Logically though, some amount of classification has to be carried out to serve as input into response selection. This is indicated by the dotted and diagonal lines within the central stages in Figure 4. Crosstalk occurs when the outcomes from one process do not only receive input from the to-be-processed task, that is, Task 1 in our case, but also from other tasks such as Task 2, which could facilitate or impede the production of the processes’ outcomes.

For both kinds of BCEs, we assume that, when S2 is presented, it is automatically classified to some degree due to its existing associations with the according category, leading to a C-R rule-mediated (transient) activation of a response for Task 2. This automatic response activation in Task 2, however, differs from the required capacity-limited processes of Task 2 stimulus classification and response selection, which can only start after the capacity-limited central stage of Task 1 (see the postponed Task 2 central stage in Figure 4). When S2 is presented concurrently with or briefly after S1, Task 2 response activation can interfere with Task 1 central processing (see Figure 4, where Task 2 response activation runs parallel to the Task 1 central stage). In the case of the R1-R2 BCE, the outcome of this activation is spatially compatible or incompatible to the outcome of the response selection process in Task 1, which leads to a shortening or lengthening of Task 1 response selection, respectively. In contrast, in the case of the S1-R2 BCE crosstalk occurs at the level of Task 1 stimulus classification. In this case, the outcome of Task 2 response activation is compatible or incompatible with the stimulus category of S1, and as a result, Task 1 stimulus classification is shortened or lengthened, respectively. It should be remembered, however, that this model is purely post-hoc and thus requires future studies to further test its value.

### Limitations, implications, and future research

Based on Sternberg’s (1969) additive factors logic we ruled out a perceptual locus of the S1-R2 BCE and reasoned that in this case, the process of Task 2 response activation might influence Task 1 stimulus classification as part of the Task 1 central stage. Strictly speaking, however, this conclusion is not inevitable. The absence of an interaction of stimulus brightness with S1-R2 compatibility is compatible with any locus, except a perceptual one. The present results could also indicate that the S1-R2 BCE has its locus within the stages of Task 1 response execution, response selection, or both. Although consistent with Sternberg’s logic, we consider these possibilities as unlikely. During S1-R2 BCE blocks, R2 had little to no overlap with R1. Specifically, whereas Task 1 required a manual left or right response, Task 2 required a vocal utterance of ‘green’ or ‘red’. These responses use different effectors as well as different response categories and there should be little if any interference. Nevertheless, we strongly encourage future research to further scrutinize our conclusion. One way to do so might once again include Sternberg’s (1969) additive factors logic. For example, manipulating the similarity between S1 stimuli likely influences the time required for stimulus classification and should thus interact with the S1-R2 BCE, but not with the R1-R2 BCE.

Finally, in the present study, we defined crosstalk based on the correspondence between S1-R2 and R1-R2. Importantly, S1 was distinct from S2 in that S2 did not apply to Task 1 S-R-Rules (i.e., the stimuli were–in terms of the task-switching literature–univalent; e.g., Kiesel et al., 2010; Koch et al., 2018). Other types of BCEs, however, can be evoked by various manipulations. Miller (2006), for example, used a go/no-go task for Task 2. When Task 2 was a no-go trial, RT1s were longer compared to when Task 2 required a go trial, which is often referred to as the *no-go BCE* (see also Janczyk & Huestegge, 2017; Ko & Miller, 2014; Röttger & Haider, 2017). Although similar to the compatibility-based BCE at a phenomenological level, recent evidence by Durst and Janczyk (2018, 2019) suggests that the no-go BCE arises due to Task 2 response selection influencing the motor execution of Task 1 (but see Röttger & Haider, 2017, for a different opinion). While this is not contradictive to our results, it certainly shows that the herein proposed mechanism for the S1-R2 and the R1-R2 BCE is specific for a certain type of BCE, namely the compatibility-based BCE. Future attempts towards a unified framework that incorporates different BCEs might be a valuable approach for dual-task research.

## Conclusion

In sum, continuing research by Janczyk et al. (2018), the present study further ruled out a perceptual locus of the S1-R2 BCE. Alternatively, we concluded that the S1-R2 BCE probably has its locus within a capacity-limited central stage, similar to the R1-R2 BCE. In detail, however, we tentatively suggest that the R1-R2 BCE affects Task 1 response selection, whereas the S1-R2 BCE influences Task 1 stimulus classification.

## Data Accessibility Statement

The data used within this study and a short description file can be found at: osf.io/86bhe.

## Footnotes

Janczyk et al. (2018; Experiments 1–3) applied the *locus of slack logic* (see Schweickert, 1978) in an extended psychological refractory period (PRP) experiment comprising three instead of two tasks. Task 1 was an unrelated binary tone discrimination task, while spatial or conceptual overlap was manipulated between Tasks 2 and 3; resulting in a BCE in Task 2. Following the locus of slack logic, an underadditive interaction of SOA with the BCE in Task 2 indicates that the BCE has its locus in a precentral stage, such as response activation or stimulus perception. Additive effects of SOA and the BCE, however, would indicate a locus of the BCE within or after the capacity-limited central stage. While all experiments revealed additive effects of SOA and the BCE, a descriptive trend towards underadditivity was indeed observed in Experiment 3, where a conceptual overlap between S2 and R3 was realized. This leaves open the possibility of a precentral locus of the S2-R3 BCE.

To ensure that our observations were not distorted by response grouping (e.g., Ulrich & Miller, 2008), we ran identical analyses excluding trials with an IRI smaller than 150, 200, or 250 ms. In none of these cases did the result pattern change.

Skeptical readers might criticize that the empirical Bayes Factors are inconclusive (with a range between 4.1 and 6.2 for Task 1). Although we agree that these values are not overwhelming, one should consider the descriptive value of the interaction between the S1-R2 BCE and stimulus brightness. The S1-R2 BCE was 57 ms and 53 ms in trials with dark compared to bright stimuli, respectively (see Table 1). The interaction thus refers to a difference of 4 ms (with a 95% confidence interval of [–13, 22]), which we consider as neglectable.

This does, of course, not deny any influence of S-R-rules, especially after extensive practice. In the present context, however, we focus on a general framework that potentially allows accounting for both the R1-R2 and the S1-R2 BCE.
